# Supplementary material for: Control points for design of taxonomic composition in synthetic human gut communities
Source: Cell Syst. 2023 Dec 20;14(12):1044–1058.e13. doi: 10.1016/j.cels.2023.11.007 (PMC10752370; doi:10.1016/j.cels.2023.11.007)
Supplement: Document S1. Figures S1–S6 and Table S1 [file mmc1.pdf]

Cell Systems, Volume *14*

Supplemental information

Control points for design of taxonomic composition in synthetic human gut communities

Bryce M. Connors, Jaron Thompson, Sarah Ertmer, Ryan L. Clark, Brian F. Pfleger, and Ophelia S. Venturelli

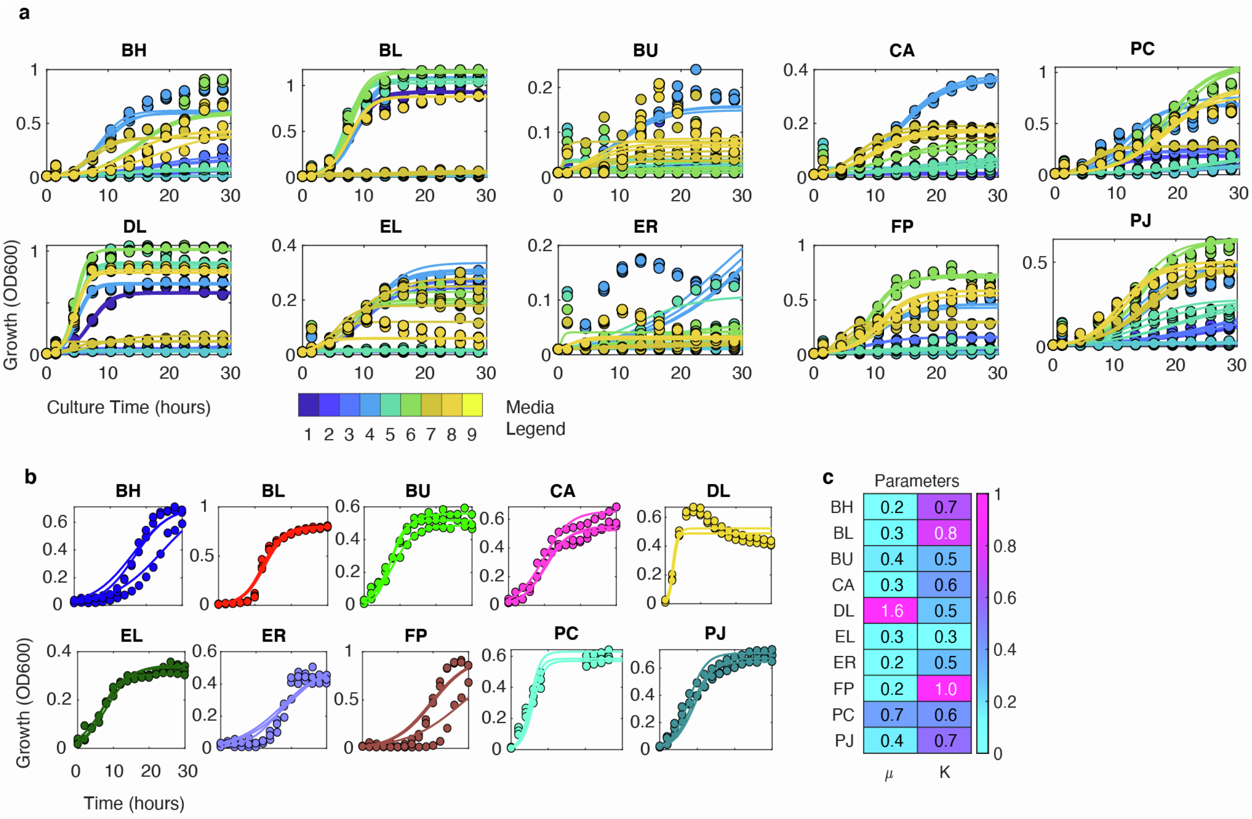
a
BH
BL
BU
CA
PC
DL
EL
ER
FP
PJ
Growth (OD600)
Culture Time (hours)
1 2 3 4 5 6 7 8 9
Media Legend
b
BH
BL
BU
CA
DL
EL
ER
FP
PC
PJ
Growth (OD600)
Time (hours)
c
Parameters
BH 0.2 0.7
BL 0.3 0.8
BU 0.4 0.5
CA 0.3 0.6
DL 1.6 0.5
EL 0.3 0.3
ER 0.2 0.5
FP 0.2 1.0
PC 0.7 0.6
PJ 0.4 0.7
μ
K

Supplementary Figure 1. Timeseries measurements and logistic model fits for media screening experiment and optimized medium related to media optimization approach detailed in Fig. 1. **(a)** Timeseries measurements of absorbance at 600 nm (OD600) (colored circles) for each monoculture (n=3-4 biological replicates). Color denotes media condition according to figure legend. Lines denote logistic model fits. The concentrations of the media components are labeled on the heatmap in Fig. 1b. All species were inoculated to 0.01 OD600. **(b)** Timeseries measurements of OD600 and logistic model fits of monocultures in the optimized medium (n=3 biological replicates). All species were inoculated at 0.01 OD600. **(c)** Heatmap of inferred logistic differential equation parameters (mean of n=3 replicate fits) for all monocultures in the optimized media: Growth rate (μ, hr^{-1}) and carrying capacity ($K, OD600$).

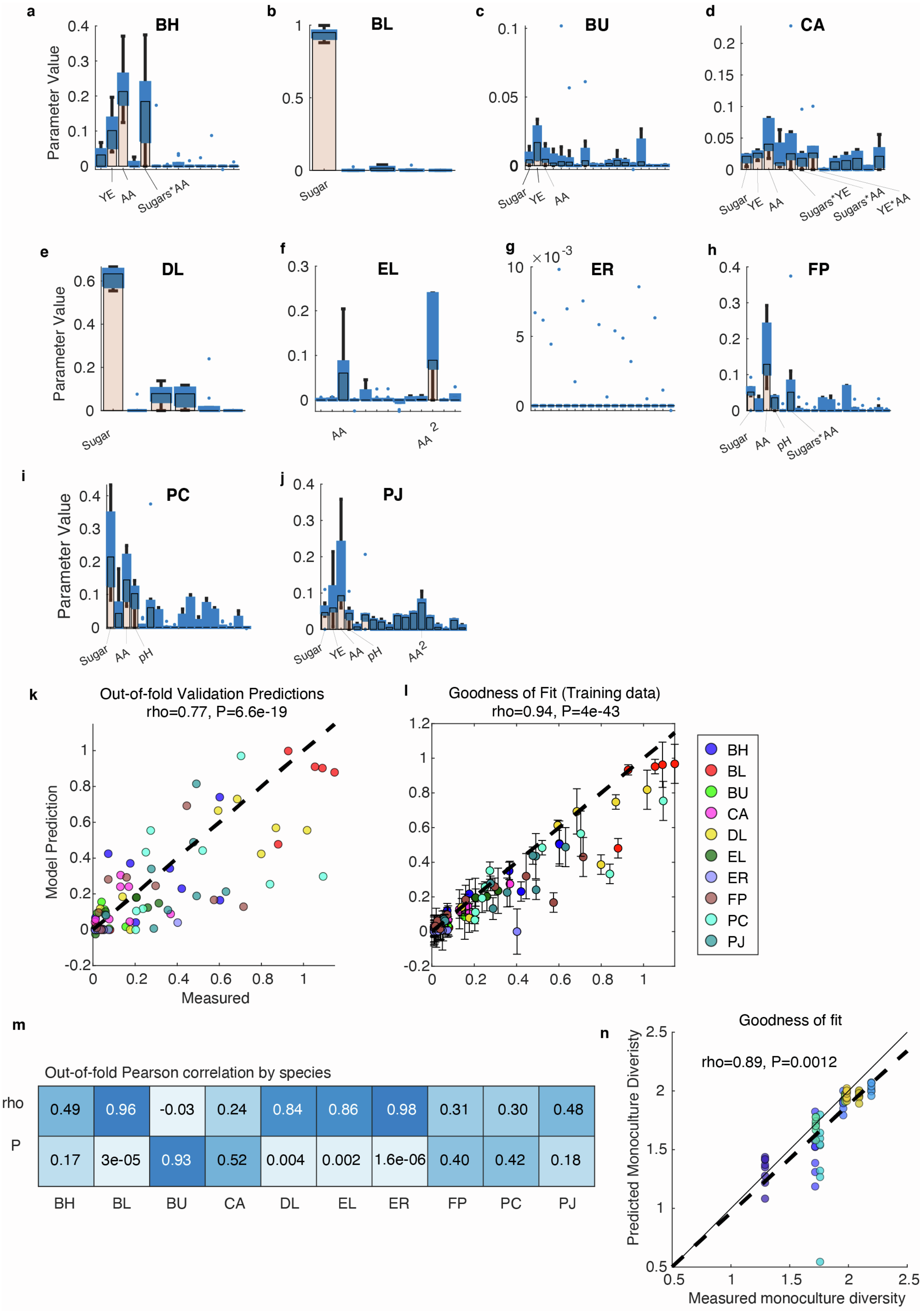
a
BH
Parameter Value
YE
AA
Sugars*AA
b
BL
Sugar
c
BU
Sugar
YE
AA
d
CA
Sugar
YE
AA
Sugars*YE
Sugars*AA
YE*AA
e
DL
Parameter Value
Sugar
f
EL
AA
AA 2
g
ER
×10 -3
h
FP
Sugar
AA
pH
Sugars*AA
i
PC
Parameter Value
Sugar
AA
pH
j
PJ
Sugar
YE
AA
pH
AA2
k
Out-of-fold Validation Predictions
rho=0.77, P=6.6e-19
Model Prediction
Measured
l
Goodness of Fit (Training data)
rho=0.94, P=4e-43
BH
BL
BU
CA
DL
EL
ER
FP
PC
PJ
m
Out-of-fold Pearson correlation by species
rho 0.49 0.96 -0.03 0.24 0.84 0.86 0.98 0.31 0.30 0.48
P 0.17 3e-05 0.93 0.52 0.004 0.002 1.6e-06 0.40 0.42 0.18
BH BL BU CA DL EL ER FP PC PJ
n
Goodness of fit
rho=0.89, P=0.0012
Predicted Monoculture Diveristy
Measured monoculture diversity

Supplementary Figure 2. Media regression model parameters and statistical evaluation related to media optimization approach detailed in Fig. 1. Boxplots indicate the distribution of parameter values for the nine leave-one-out parameter sets for media regression models (MR, Table S1) for **(a)** BH**, (b)** BL**, (c)** BU, (**d**) CA, (**e**) DL, (**f**) EL, (**g**) ER, (**h**) FP, (**i**) PC or (**j**) PJ. Bar height indicates median value of the given regression model parameter, box upper and lower edges indicate 75th and 25th percentiles, whiskers represent range. Labels corresponding to largest parameter values are shown. Parameters with a value of zero across all sets are not plotted. Many parameter values are driven to zero by elastic net regularization. All models consist of linear regression with linear and quadratic main effects terms, and 2nd and 3rd order interaction terms. Predictors (independent variables) are scaled between 0 and 1 (corresponding to low and high experimental design conditions, respectively) prior to parameter inference, while growth data is not scaled to allow comparison between species. The value on the y-axis represents the sensitivity of each specie's predicted growth to a change in the concentration of the respective media variable. For example, a large "sugar" parameter indicates that this metabolite has a strong inferred effect on a given specie's growth. **(k)** Scatter plot of out-of-fold predictions for the ten media regression models predicting the growth of each species. Nested cross validation is used to fit nine "leave-one-out" parameter sets (Methods). Pearson correlation (rho) and p-value (P) for all out-of-fold predictions. **(l)** Scatter plot of model predictions on training data (in-fold predictions). Colored circles represent mean across eight in-fold predictions (i.e., predictions on training data) and error bars indicate 1 s.d. from the mean. Goodness of fit statistics (Pearson rho and P) are calculated between the measured data and the mean of the ensemble prediction distribution on training data. **(m)** Heatmap of Pearson correlations and corresponding p-values for out-of-fold model predictions for each species. We note that the high correlation for ER despite inconsistent model parameters is a statistical artifact of many predictions near zero and one non-zero prediction (i.e., effectively fitting a line to two points). **(n)** Goodness of fit scatter plot of predicted vs. measured monoculture-diversity. Goodness of fit is taken between the average of the monoculture-diversities calculated from regression model predictions on training data (e.g. across 8 points of a similar color) and the monoculture-diversity calculated from the inferred carrying capacities. Model predictivity is evaluated in Fig. 1f, wherein carrying capacity regression model predictions of test data are used to calculate monoculture-diversity. Pearson correlation coefficient (rho) and p-value (P) are indicated.

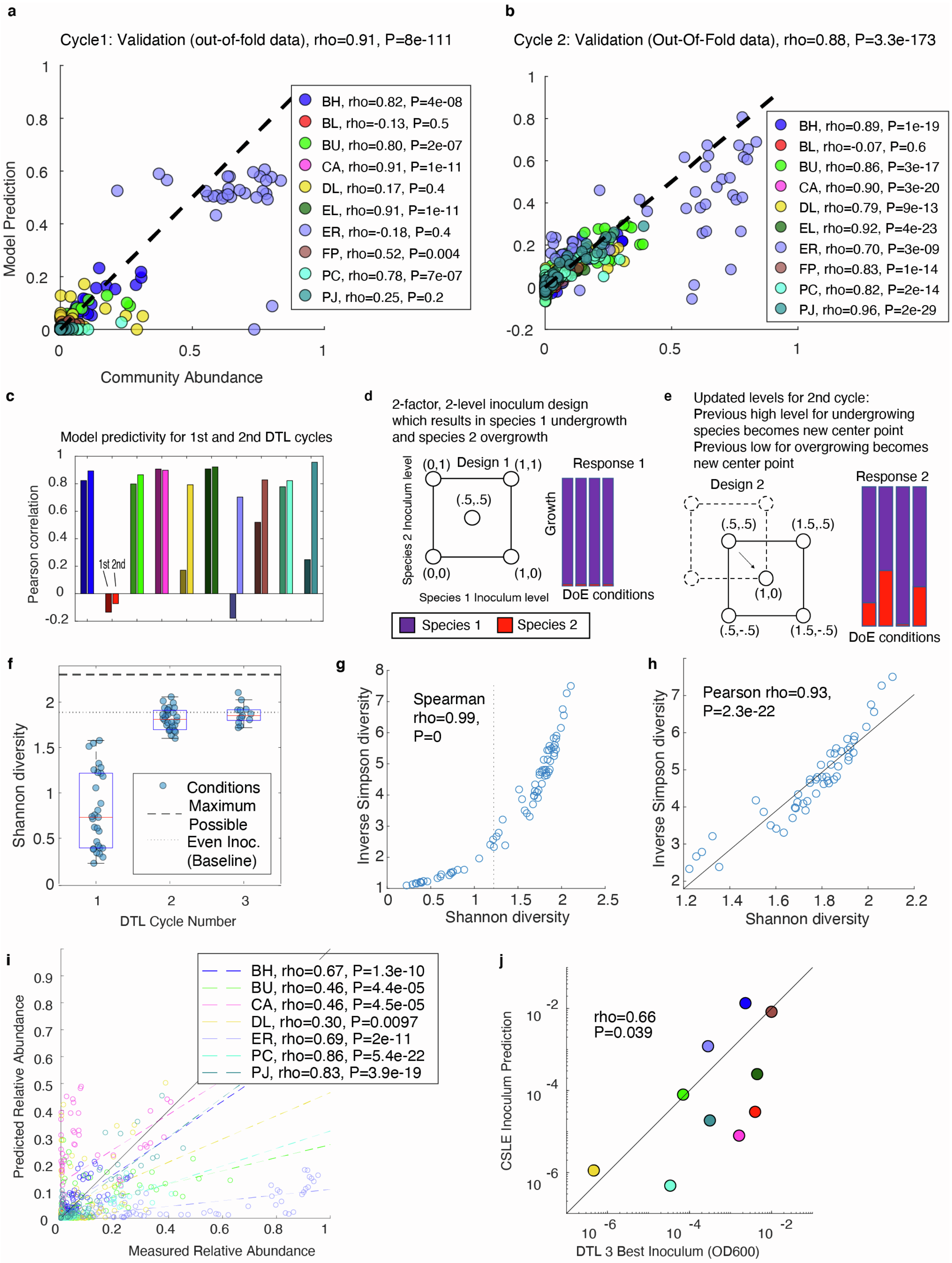

a
Cycle1: Validation (out-of-fold data), rho=0.91, P=8e-111
Model Prediction
Community Abundance
BH, rho=0.82, P=4e-08
BL, rho=-0.13, P=0.5
BU, rho=0.80, P=2e-07
CA, rho=0.91, P=1e-11
DL, rho=0.17, P=0.4
EL, rho=0.91, P=1e-11
ER, rho=-0.18, P=0.4
FP, rho=0.52, P=0.004
PC, rho=0.78, P=7e-07
PJ, rho=0.25, P=0.2
b
Cycle 2: Validation (Out-Of-Fold data), rho=0.88, P=3.3e-173
BH, rho=0.89, P=1e-19
BL, rho=-0.07, P=0.6
BU, rho=0.86, P=3e-17
CA, rho=0.90, P=3e-20
DL, rho=0.79, P=9e-13
EL, rho=0.92, P=4e-23
ER, rho=0.70, P=3e-09
FP, rho=0.83, P=1e-14
PC, rho=0.82, P=2e-14
PJ, rho=0.96, P=2e-29
c
Model predictivity for 1st and 2nd DTL cycles
Pearson correlation
1st 2nd
d
2-factor, 2-level inoculum design
which results in species 1 undergrowth
and species 2 overgrowth
Design 1
Response 1
(0,1) (1,1) (.5,.5) (0,0) (1,0)
Species 2 Inoculum level
Species 1 Inoculum level
Growth
DoE conditions
Species 1
Species 2
e
Updated levels for 2nd cycle:
Previous high level for undergrowing
species becomes new center point
Previous low for overgrowing becomes
new center point
Design 2
Response 2
(.5,.5) (1.5,.5) (1,0) (.5,-.5) (1.5,-.5)
DoE conditions
f
Shannon diversity
DTL Cycle Number
Conditions
Maximum Possible
Even Inoc. (Baseline)
g
Spearman rho=0.99, P=0
Inverse Simpson diversity
Shannon diversity
h
Pearson rho=0.93, P=2.3e-22
Inverse Simpson diversity
Shannon diversity
i
Predicted Relative Abundance
Measured Relative Abundance
BH, rho=0.67, P=1.3e-10
BU, rho=0.46, P=4.4e-05
CA, rho=0.46, P=4.5e-05
DL, rho=0.30, P=0.0097
ER, rho=0.69, P=2e-11
PC, rho=0.86, P=5.4e-22
PJ, rho=0.83, P=3.9e-19
j
rho=0.66
P=0.039
CSLE Inoculum Prediction
DTL 3 Best Inoculum (OD600)

Supplementary Figure 3. Regression models and additional information for DTL cycles related to inoculation density optimization detailed in Fig. 3**. (a)** Scatter plot of inoculum regression model predictions (IR1, models trained on DTL cycle 1 data only, Table S1) vs. experimental measurements (mean of n=3 biological replicates) for out-of-fold conditions and predictions. Models are trained on absolute abundance estimates calculated as the product of specie's relative abundance by OD600. Pearson correlation (rho) and p-value (P) for each species are indicated in the legend. **(b)** Scatter plot of inoculum regression model predictions (IR2, models trained on DTL cycle 1+2 data, Table S1) vs. experimental measurements (mean of n=3 biological replicates) for out-of-fold predictions. Pearson correlations and p-values for each species are indicated in the legend. **(c)** Bar plot denotes Pearson correlation coefficients after adding DTL 2 training data (IR1 vs. IR2, Table S1). Darker left-hand bars indicate DTL 1 Pearson correlation coefficients (numerical values shown in panel "a" legend). Lighter, right-hand bars indicate DTL 2 Pearson correlation coefficients (panel b legend). **(d)** Cartoon schematic depicting empirical design approach of new inoculation densities used when model performance was inadequate (Methods). A hypothetical two-factor (species), two-level (inoculum density) design with center point. This hypothetical experimental design yields community compositions in which the growth responses of both species 1 and 2 are not affected by the design levels: Species 1 due to lower than measurable growth, species 2 due to saturating overgrowth. Since the responses are not measurably correlated with the design variables, a predictive model cannot be inferred. We reason that if a species exceeded target composition, we should decrease the inoculum density, and vice versa. **(e)** We update the inoculation levels by a half "frameshift" of the previous design levels (i.e. shifting the range of setpoints by half of its value in the desired direction). In other words, the new design uses the extremum of the old design as its new center point (low if overgrowth, high if undergrowth). Half of the new design thus overlaps with the old design, and the new "center point" level (previous extrema) can be used as an input regression models that were predictive, without forcing the models to extrapolate beyond training data. This approach flexibly integrates both experimental intuition and model-guided design. **(f)** Distributions of Shannon diversities calculated from the mean composition of biological replicates (n=3) for conditions of each DTL cycle (blue circles). Red line in each box denotes the median, upper and lower edges denote 75th and 25th percentiles, respectively, and whiskers denote range of non-outlier datapoints. Dashed line indicates maximum possible Shannon diversity for a 10-member community. Dotted line indicates the diversity from even inoculum in the optimized medium. **(g)** Scatter plot of Shannon diversities across DTL cycles (per panel f) vs. inverse Simpson diversity. Dotted line indicates Shannon diversity of baseline condition. Spearman rank order correlation and p-value are indicated by rho and P, respectively. **(h)** Scatter plot of Shannon diversities greater than baseline condition vs. corresponding inverse Simpson diversity. The near linear relationship (Pearson rho, P indicates P-value) suggests that maximization of Shannon diversity also maximizes the inverse Simpson diversity in this range of interest. **(i)** Scatter plot of CLSE model predictions of relative abundances for DTL cycle communities (Fig. 3d). The seven species with a statistically significant relationship between model predictions and community abundance are shown. Though it is likely impossible to predict community assembly from monoculture data alone, this figure suggests that the constrained logistic model informed by monoculture data has an informative relationship with certain specie's abundances in the community. CSLE model parameters in this panel were inferred from monoculture data alone demonstrating K_{comm} can be inferred rather than assigned empirically (Methods). **(j)** Log-log scatter plot of the set of inoculum densities yielding best community Shannon diversity vs. inoculum densities predicted by the CLSE model to maximize Shannon diversity. Inoculum densities are log-transformed throughout the manuscript (e.g. Fig. 2c, 3b, DTL design levels in **Table S3**) because they span many orders of magnitude. As such, the Pearson correlation (rho) is calculated on the log transformed values. Diagonal line indicates x=y. This significant correlation supports the idea that the CSLE model was useful for leveraging monoculture information to estimate community assembly and inform a smaller design space in which to conduct community experiments.

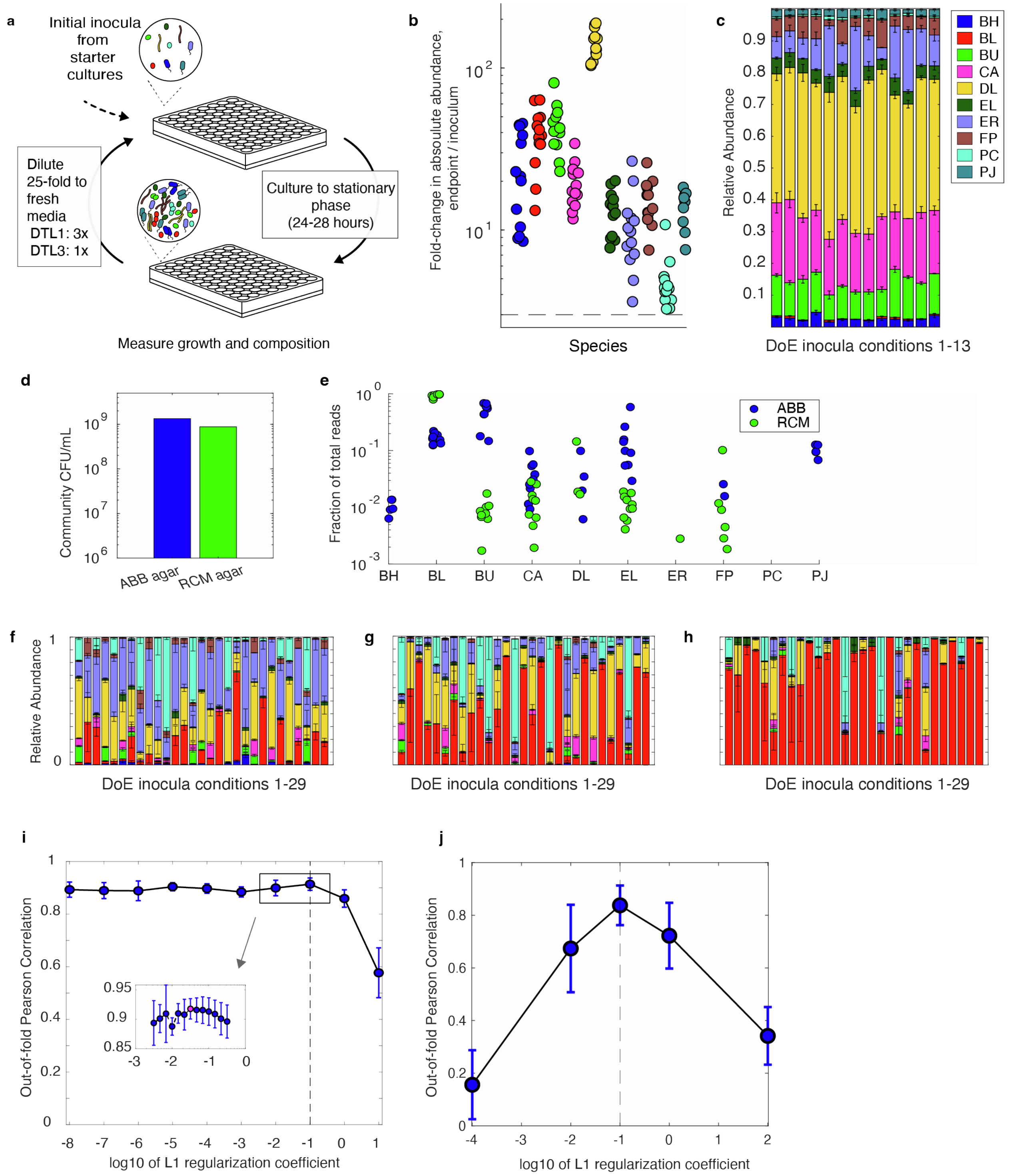
a
Initial inocula from starter cultures
Dilute 25-fold to fresh media
DTL1: 3x
DTL3: 1x
Culture to stationary phase (24-28 hours)
Measure growth and composition
b
Fold-change in absolute abundance, endpoint / inoculum
Species
c
Relative Abundance
BH
BL
BU
CA
DL
EL
ER
FP
PC
PJ
DoE inocula conditions 1-13
d
Community CFU/mL
ABB agar
RCM agar
e
Fraction of total reads
ABB
RCM
BH
BL
BU
CA
DL
EL
ER
FP
PC
PJ
f
g
h
Relative Abundance
DoE inocula conditions 1-29
DoE inocula conditions 1-29
DoE inocula conditions 1-29
i
j
Out-of-fold Pearson Correlation
log10 of L1 regularization coefficient
Out-of-fold Pearson Correlation
log10 of L1 regularization coefficient

Supplementary Figure 4. DTL passaging data used for gLV training and evaluation related to DTL viability (Fig. 3) and gLV modeling (Fig. 4) **. (a)** Schematic of community passaging experiments. Experiments were performed by diluting (25-fold volume/volume ratio) an aliquot of the stationary phase community cultures into fresh media, and then culturing these communities again until stationary phase. Measurements of community growth (OD600) and pellets for 16S rRNA gene next-generation sequencing are collected in the stationary phase of each passage. (**b)** Categorial scatter plot of the fold-change in the endpoint absolute abundance estimates of each species divided by its corresponding inoculum absolute abundance for DTL 3, passage 2. The endpoint abundance of each species was greater than 3-fold (denoted by dashed line) compared to their initial abundance. (**c**) Stacked bar plots show the relative abundance (bar height denotes mean, error bars 1 s.d. from the mean of n=3 biological replicates) for the additional passage of DTL 3. Biological replicates are plotted individually in Fig. S6l. **(d)** Bar plot indicates colony forming units per milliliter for 100 mL community cultured from the best inoculation condition (Fig. 3h). Bar height indicates the average across six technical replicates cultured on anaerobic basal broth (ABB) agar plates or reinforced clostridial media (RCM) agar plates. **(e)** Fraction of total reads of each species from pooled colonies of each type of agar plate. Like colored points represent distribution of read fractions across six technical replicates for each of two serial dilution levels. These data demonstrate that the community contained viable populations of at least 9 of 10 species but do not indicate quantification of viable relative abundances due to many potential biases in the method (e.g., colony size, growth rate). PC displayed growth in several conditions of a passage of DTL 3 communities (panels b,c), suggesting that viable populations of this organism were present but not detected by the CFU plate scrape method. (**f-h**) Bar plot of community compositions for passages 2-5 of DTL 1 used for gLV training. Biological replicates are plotted individually in Fig. S6j-k. (**i**) Scatter plot indicating out-of-fold model accuracy (Pearson correlation) vs. regularization coefficient value (lambda) for 90% of full community dataset used for cross validation (Methods). The full dataset is comprised of 201 community samples each containing abundance of 10 species averaged across n=3 biological replicates. Approximately 181 samples (90%) are then used in the cross-validation procedure yielding five training partitions of about 145 samples. The best regularization coefficient identified during cross validation is used to infer a new parameter set from the original 90%, and the predictive capability of this model is evaluated on the withheld 10% (Fig. 4b). Circles indicate mean value and error bars indicate 1 s.d. across data partitions for 5-fold cross validation. The larger plot indicates a wider range of coefficients initially tested, while the smaller inlayed plot depicts a higher resolution sampling of coefficient values near the best value identified in the coarse sampling. The L1 regularization coefficient that maximizes the average out-of-fold Pearson correlation coefficient during cross validation and is indicated by the dashed line and pink circle. (**j**) Scatter plot indicating out-of-fold model accuracy (Pearson correlation) vs. regularization coefficient value (lambda) for 25% of full community dataset used for cross validation (Methods). The presence of a strong maximum in correlation coefficient for the smaller training dataset in comparison to high predictivity over a range of correlation coefficients for the larger dataset (Fig. S4i) suggests that the larger dataset is sufficient to constrain the model without regularization while the overfitting of parameters to smaller datasets is mitigated by regularization.

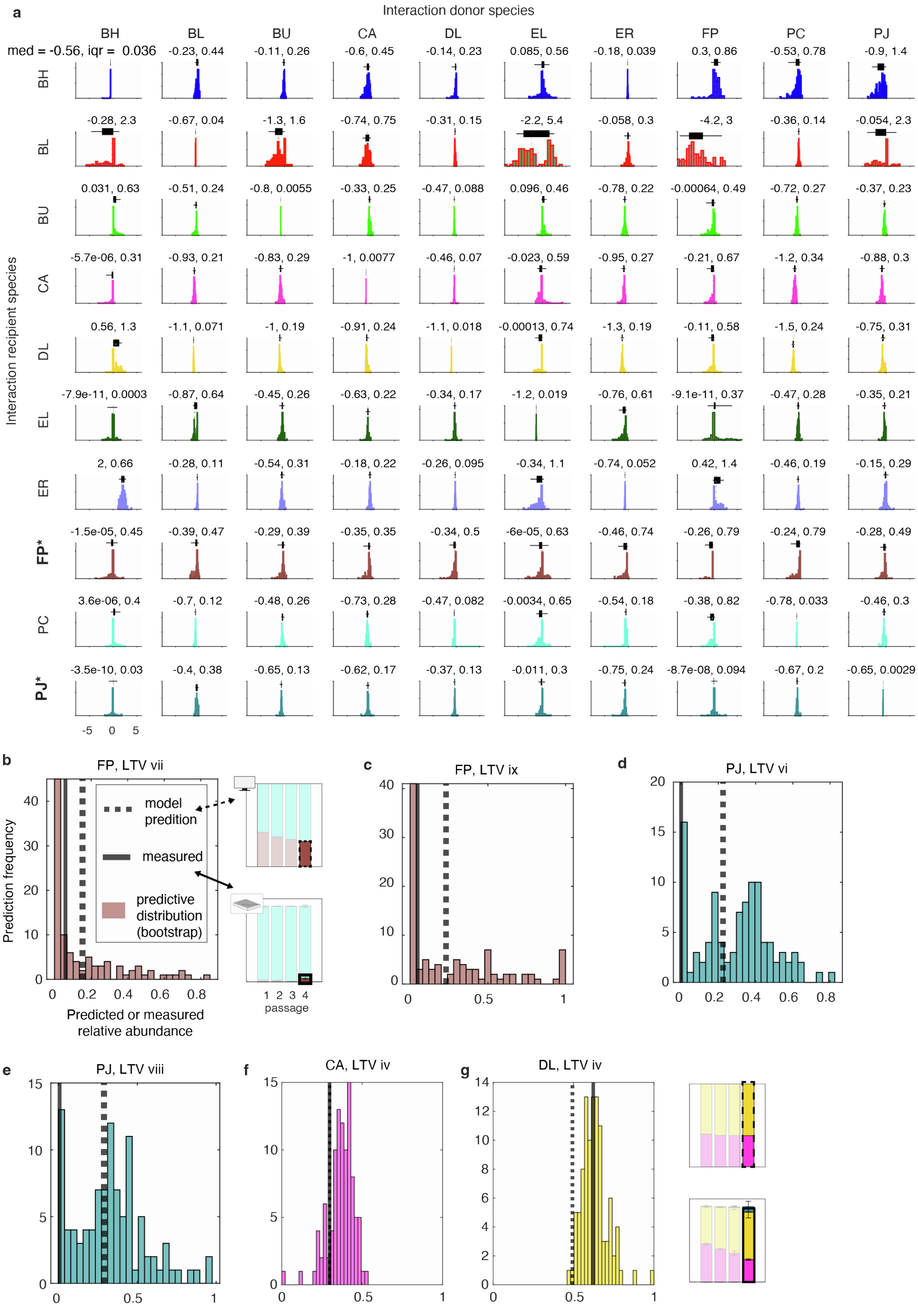
a
Interaction donor species
BH BL BU CA DL EL ER FP PC PJ
med = -0.56, iqr = 0.036
-0.23, 0.44
-0.11, 0.26
-0.6, 0.45
-0.14, 0.23
0.085, 0.56
-0.18, 0.039
0.3, 0.86
-0.53, 0.78
-0.9, 1.4
-0.28, 2.3
-0.67, 0.04
-1.3, 1.6
-0.74, 0.75
-0.31, 0.15
-2.2, 5.4
-0.058, 0.3
-4.2, 3
-0.36, 0.14
-0.054, 2.3
0.031, 0.63
-0.51, 0.24
-0.8, 0.0055
-0.33, 0.25
-0.47, 0.088
0.096, 0.46
-0.78, 0.22
-0.00064, 0.49
-0.72, 0.27
-0.37, 0.23
-5.7e-06, 0.31
-0.93, 0.21
-0.83, 0.29
-1, 0.0077
-0.46, 0.07
-0.023, 0.59
-0.95, 0.27
-0.21, 0.67
-1.2, 0.34
-0.88, 0.3
0.56, 1.3
-1.1, 0.071
-1, 0.19
-0.91, 0.24
-1.1, 0.018
-0.00013, 0.74
-1.3, 0.19
-0.11, 0.58
-1.5, 0.24
-0.75, 0.31
-7.9e-11, 0.0003
-0.87, 0.64
-0.45, 0.26
-0.63, 0.22
-0.34, 0.17
-1.2, 0.019
-0.76, 0.61
-9.1e-11, 0.37
-0.47, 0.28
-0.35, 0.21
2, 0.66
-0.28, 0.11
-0.54, 0.31
-0.18, 0.22
-0.26, 0.095
-0.34, 1.1
-0.74, 0.052
0.42, 1.4
-0.46, 0.19
-0.15, 0.29
-1.5e-05, 0.45
-0.39, 0.47
-0.29, 0.39
-0.35, 0.35
-0.34, 0.5
-6e-05, 0.63
-0.46, 0.74
-0.26, 0.79
-0.24, 0.79
-0.28, 0.49
3.6e-06, 0.4
-0.7, 0.12
-0.48, 0.26
-0.73, 0.28
-0.47, 0.082
-0.0034, 0.65
-0.54, 0.18
-0.38, 0.82
-0.78, 0.033
-0.46, 0.3
-3.5e-10, 0.03
-0.4, 0.38
-0.65, 0.13
-0.62, 0.17
-0.37, 0.13
-0.011, 0.3
-0.75, 0.24
-8.7e-08, 0.094
-0.67, 0.2
-0.65, 0.0029
Interaction recipient species
BH BL BU CA DL EL ER FP* PC PJ*
-5 0 5
b
FP, LTV vii
Prediction frequency
model predition
measured
predictive distribution (bootstrap)
Predicted or measured relative abundance
1 2 3 4
passage
c
FP, LTV ix
d
PJ, LTV vi
e
PJ, LTV viii
f
CA, LTV iv
g
DL, LTV iv

Supplementary Figure 5. Bootstrap analysis of gLV model uncertainty related to Fig. 4 and model guided design of dynamics (Fig. 5)**.** (**a**) Histograms of gLV interaction parameter distributions from bootstrap analysis of model uncertainty (Methods). Median parameter value and interquartile ranges are shown above each panel. A boxplot above each distribution indicates the median value, 25th and 75th percentiles (box), and 5th and 95th percentiles (whiskers). The bold fond and asterisks on the row labels of FP and PJ denote that these species were poorly predicted by point estimates of the model in multiple designed temporal variability communities (Fig. 5e communities vi-ix). Recipient species corresponds to row label, donor species corresponds to column label. Self-interaction terms fall on the diagonal and are well constrained by monoculture timeseries data. Distributions correspond to resampling the original training set (consisting of 90% of the full community dataset, randomly sampled) 100 times (Methods). All x-axes are identically scaled per tick labels on lower left subplot. (**b-e**) Histogram of bootstrapped predictive distributions for the two species with multiple qualitatively incorrect predictions in the designed low temporal variability communities (Fig. 5e). Distributions are for final passage relative abundance predictions of (**b**) FP vii, (**c**) FP, ix, (**d**) PJ vi, (**e**) viii, as indicated by the stacked bar plot in panel in panel b. Final passage distributions are representative of earlier passages in all cases. Dashed line represents the prediction of the gLV model, corresponding to the stacked bar plot segment outlined with a dashed line. Solid line indicates the measured relative abundance during experimental validation (mean of n=3 biological replicates), and corresponds to the stacked bar plot segment outlined with a solid line. **f,g** Histogram of bootstrapped predictive distributions for CA (f) and DL (g) in community iv, which had comparatively high confidence and was well predicted by the original model.

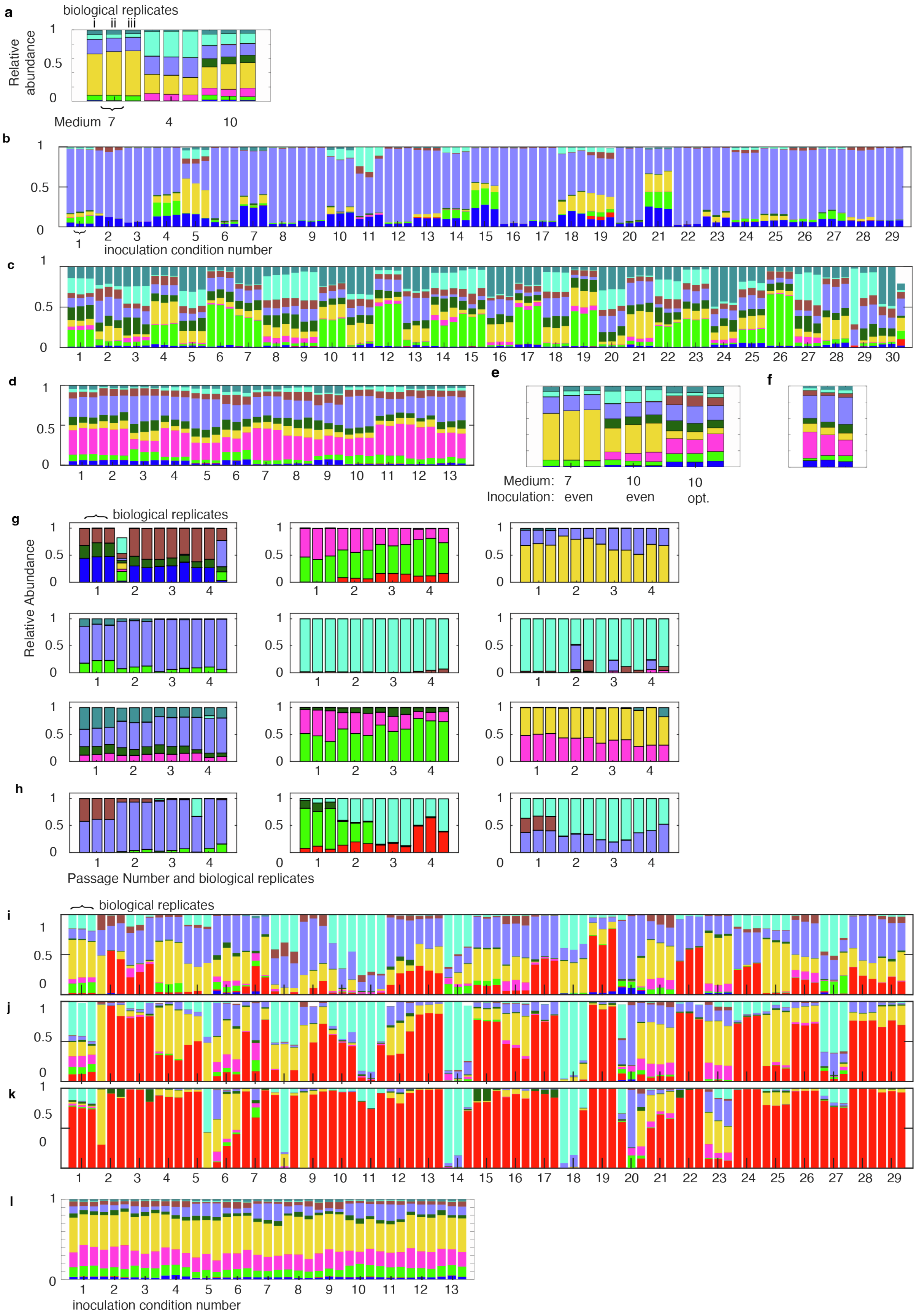
a
biological replicates
i ii iii
Relative abundance
Medium 7 4 10
b
inoculation condition number
c
d
e
Medium: 7 10 10
Inoculation: even even opt.
f
g
biological replicates
Relative Abundance
h
Passage Number and biological replicates
i
biological replicates
j
k
l
inoculation condition number

Supplementary Figure 6. Biological replicates for compositional data related to Figs. 1,3, and 5 containing stacked bar plots of community composition. Main text figures show mean and standard deviation of n=3 biological replicates to compactly visualize large datasets; in this figure each replicate is plotted individually. X-axes show n=3 replicates, plotted adjacently, for each experimental. All y-axes are scaled between 0 and 1. Biological replicates correspond to the following figures: **a** Fig. 1i where x-axis labels denote media type for adjacent sets of replicates, **b-d** Fig. 3d DTL1-3, respectively, where x-axis labels denote designed inoculation condition number for adjacent sets of replicates, **e** Fig. 3f where x-axis labels denote media and inoculation conditions for sets of adjacent biological replicates, **f** Fig. 3g where x-axis shows biological replicates of 100mL scale up condition. Replicates of the 200uL condition are shown in panel d, condition 13. **g,h** Fig. 5e,d where x labels denote passage number of adjacent sets of replicates, **i-k** Fig. S4 d-f where x-axis labels denote original inoculation conditions for sets of adjacent biological replicates, and **l** S4c where x-axis labels denote original inoculation conditions for sets of adjacent biological replicates.

	**Species Name**	**Strain Name**	**Source**	**Inoculum Volume from glycerol stock (uL)**	**Pre-culture Media**	**Pre-culture Time (hr)**
BH	*Blautia hydrogenotrophica*	*Blautia hydrogenotrophica* S5a33 [DSM 10507]	DSMZ	400	OM	24
BL	*Bifidobacterium longum subs. infantis*	*Bifidobacterium longum subs. infantis* S12 [DSM 20088]	DSMZ	400	1:1 OM:ABB	40
BU	*Bacteroides uniformis*	*Bacteroides uniformis* VPI 0061 [DSM 6597]	DSMZ	150	OM	24
CA	*Collinsella aerofaciens*	*Collinsella aerofaciens* VPI 1003 [DSM 3979]	DSMZ	400	OM	40
DL	*Dorea longicatena*	*Dorea longicatena* 111-35	Rey Lab	150	1:1 OM:ABB	24
EL	*Eggerthella lenta*	*Eggerthella lenta* 1899 B [DSM 2243]	DSMZ	400	1:1 OM:ABB	24
ER	*Eubacterium rectale*	*Eubacterium rectale* VPI 0990 [ATCC 33656]	ATCC	400	1:1 OM:ABB	40
FP	*Faecalibacterium prausnitzii*	*Faecalibacterium prausnitzii* A2-165 [DSM 17677]	DSMZ	400	OM	40
PC	*Prevotella copri*	*Prevotella copri* CB7 [DSM 18205]	DSMZ	400	1:1 OM:ABB	24
PJ	*Parabacteroides johnsonii*	*Parabacteroides johnsonii* M-165 [DSM 18315]	DSMZ	150	ABB	24
ABB - 5 mL Anaerobic Basal Broth (Oxoid)<br>OM - 5 mL optimized medium at pH 6.7<br>1:1 OM:ABB - 2.5 mL of OM + 2.5 mL of ABB						

Table S1. Strain sources and preculturing conditions for synthetic community assembly, related to Figure 1.
